# Transcriptome-based analysis of mitogen-activated protein kinase cascades in the rice response to *Xanthomonas oryzae* infection

**DOI:** 10.1186/s12284-014-0038-x

**Published:** 2015-01-27

**Authors:** Zeyu Yang, Haigang Ma, Hanming Hong, Wen Yao, Weibo Xie, Jinghua Xiao, Xianghua Li, Shiping Wang

**Affiliations:** National Key Laboratory of Crop Genetic Improvement, National Center of Plant Gene Research (Wuhan), Huazhong Agricultural University, Wuhan, 430070 China

**Keywords:** Bacterial blight, Defense, Disease, Mitogen-activated protein kinase, *Oryza sativa*

## Abstract

**Background:**

Mitogen-activated protein (MAP) kinase cascades, with each cascade consisting of a MAP kinase kinase kinase (MAPKKK), a MAP kinase kinase (MAPKK), and a MAP kinase (MAPK), play important roles in dicot plant responses to pathogen infection. However, no single MAP kinase cascade has been identified in rice, and the functions of MAP kinase cascades in rice − pathogen interactions are unknown.

**Results:**

To explore the contribution of MAP kinase cascade in rice in response to *Xanthomonas oryzae* pv. *oryzae* (*Xoo*), which causes bacterial blight, one of the devastating diseases of rice worldwide, we performed a comprehensive expression analysis of rice MAP kinase cascade genes. We transcriptionally analyzed all the 74 MAPKKK genes, 8 MAPKK, and 17 MAPK genes in two pairs of susceptible and resistant rice lines, with each pair having the same genetic background, to determine the rice response to *Xoo* infection. The expression of a large number of MAP kinase cascade genes changed in response to infection, and some of the genes also showed different expression in resistant and susceptible reactions. In addition, some MAPKKK genes co-expressed with MAPKK and/or MAPK genes, and MAPKK genes co-expressed with MAPK genes.

**Conclusions:**

These results provide a new perspective regarding the putative roles of rice MAP kinase gene candicates and potential cascade targets for further characterization in rice–pathogen interactions.

**Electronic supplementary material:**

The online version of this article (doi:10.1186/s12284-014-0038-x) contains supplementary material, which is available to authorized users.

## Background

Mitogen-activated protein kinase (MAP) kinase cascades play pivotal roles in the signaling of diverse developmental and physiological processes of plants, including host–pathogen interactions. Each MAP kinase cascade consists of at least three evolutionarily conserved signaling molecules, MAP kinase kinase kinase (MAPKKK), MAP kinase kinase (MAPKK), and MAP kinase (MAPK) (Tena et al. 2001). MAPKKK phosphorylates MAPKK and MAPKK phosphorylates MAPK, which in turn phosphorylates downstream components leading to the activation or suppression of a signaling pathway. The three types of protein kinases each belong to a family. Each of these protein kinases can be the component of more than one MAP kinase cascade, and the same signal or biological activity may be regulated by different MAP kinase cascades or cross-linked MAP kinase cascades (Rasmussen et al. 2012).

MAP kinase signaling has been reported to be involved in both pathogen-associated molecular pattern–triggered immunity (PTI), or basal resistance, and effector-triggered immunity (ETI), or race-specific resistance (Meng and Zhang 2013). In plant defense responses against pathogens, a member of the MAP kinase cascade can be a positive regulator or a negative regulator; for example, a MAP kinase cascade consisting of MEKK1 (a MAPKKK), MKK4/MKK5 (two redundant MAPKKs), and MPK3/MPK6 (two redundant MAPKs) positively regulates the *Arabidopsis* PTI trigger by bacterial flg22 (Asai et al. 2002; Ren et al. 2002). Another *Arabidopsis* MAP kinase cascade consisting of MEKK1, MKK1/MKK2 (two redundant MAPKKs), and MPK4 (a MAPK) negatively regulates both flg22-triggerred PTI and the nucleotide-binding leucine-rich repeat (NB-LRR) protein SUMM2–initiated ETI (Petersen et al. 2000; Ichimura et al. 2006; Nakagami et al. 2006; Suarez-Rodriguez et al. 2007; Qiu et al. 2008; Kong et al. 2012; Zhang et al. 2012). The positive and negative roles of these two MAP kinase cascades are attributable to the MPK3/MPK6 promoting the defense response and MPK4 suppressing the defense response (Petersen et al. 2000; Asai et al. 2002). A MAP kinase cascade consisting of MAPKKKα, NtMEK2, and SIPK/WIPK (two redundant MAPKs) regulates the immunity in tobacco (Jin et al. 2003; del Pozo et al. 2004).

Rice genome contains 74 MAPKKK genes (*MPKKK36* and *MPKKK56* are the same gene), 8 MAPKK genes, and 17 MAPK genes (Hamel et al. 2006; Reyna and Yang 2006; Rao et al. 2010). The markedly different numbers of the three types of proteins suggest that MAP kinase cascades initiated with different MAPKKK may share the same MAPKK or MAPK, or that a MAPKK or MAPK may be involved in multiple biological activities. The large numbers of MAPKKK in the rice genome also suggest that MAP kinase cascades may be important for many physiological processes. However, the importance of MAP kinase cascades in pathogen-induced rice defense signaling cannot be evaluated because only three rice MAPKs and one rice MAPKKK have been confirmed to be involved in rice–pathogen interactions so far. The MPK5/OsMAPK5 (named OsMAPK5 in Xiong and Yang 2003) negatively regulates rice resistance to fungal *Magnaporthe oryzae* and bacterial pathogens *Burkholderia glumae* and *Xanthomonas oryzae* pv. *oryzae* (*Xoo*) (Seo et al. 2011). Rice MPK6 plays opposite roles in the rice response to *Xoo* infection; it positively regulates local resistance but negatively regulates systemic acquired resistance after *Xoo* invasion (Yuan et al. 2007; Shen et al. 2010). Rice MPK12 positively regulates resistance to *Xoo* (Seo et al. 2011). The MPKKK1/OsEDR1 (named OsEDR1 in Shen et al. 2011) negatively regulates rice resistance to *Xoo* and positively regulates rice resistance to *M. oryzae*.

*Xoo* causes bacterial blight, which is one of the most devastating diseases of rice worldwide (Kou and Wang 2013). The NB-LRR protein–initiated ETI is not the major type of qualitative resistance to *Xoo* in rice; therefore, rice–*Xoo* interaction provides a unique pathosystem to study the diverse mechanisms of host resistance (Zhang and Wang 2013). To facilitate the characterization of genes and entire MAP kinase cascades putatively involved in the rice response to pathogen infection, we transcriptionally analyzed all the MAPKKK, MAPKK, and MAPK genes in rice–*Xoo* interactions in two pairs of susceptible and resistant rice lines. The each pair of rice lines had the same genetic background, but the resistant lines in each pair carried different types of major disease resistance (*MR*) gene, which confers qualitative resistance, either a ETI or a PTI (Zhang and Wang 2013). We also conducted co-expression analyses of these genes to discover potential MAP kinase cascades. Our results provide a comprehensive perspective for examining the MAP kinase signaling network and the putative roles of MAP kinase cascade genes in defense responses against bacterial pathogens. These results also provide gene targets and candidate MAP kinase cascades for further studies.

## Results

### Anatomical/developmental classification of MAP kinase cascade genes

**Figure 1:**
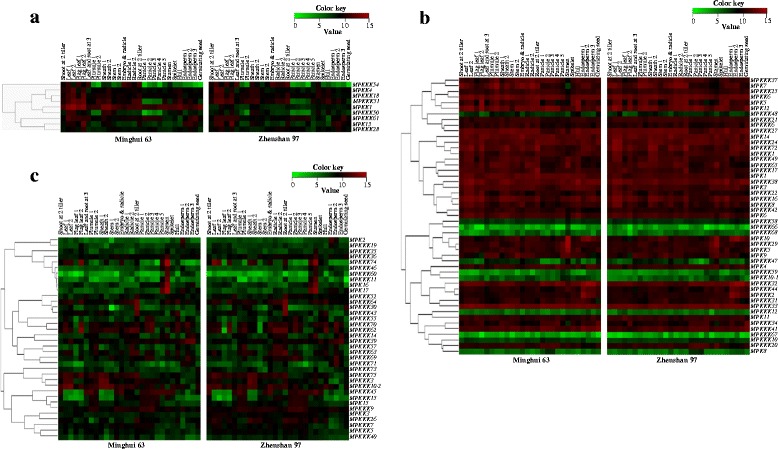
**Expression profiles of MAP kinase cascade genes including 70** ***MPKKK*** **s, 7** ***MPKK*** **s, and 17** ***MPK*** **s in 28 tissues covering the entire life cycle of indica rice varieties Minghui 63 and Zhenshan 97.** Data were obtained from a microarray database (http://www.ncbi.nlm.nih.gov; accession number GSE19024; Wang et al. 2010). Shoot at 2 tiller, shoot of seedling with two tillers; Leaf 1 and 2, at secondary branch primordium stage and 4-cm to 5-cm young panicle stage, respectively; Flag leaf 1 and 2, at 5 days before heading and 14 days after heading, respectively; Leaf and root at 3, at three-leaf stage; Plumule 1 and 2, at 48 hours after emergence under light and dark, respectively; Sheath 1 and 2, at secondary branch primordium stage and 4-cm to 5-cm young panicle stage, respectively; Stem 1 and 2, at 5 days before heading and heading stage, respectively; Embryo & radicle, embryo and radicle at 3 days after germination; Radicle 1 and 2, at 48 hours after emergence under light and dark, respectively; Root at 2 tiller, root of seedling with two tillers; Panicle 1, 2, 3, 4 and 5, at secondary branch primordium stage, pistil and stamen primordium differentiation stage, pollen–mother cell formation stage, 4-cm to 5-cm young panicle stage and heading stage, respectively; Stamen, at 1 day before flowering; Spikelet, at 3 days after pollination; Hull, at 1 day before flowering; Endosperm 1, 2 and 3, at 14 days after heading, 7 days after pollination and 21 days after pollination, respectively; Geminating seed, germinating seed at 72 hours of imbibitions. Expression levels (log2 transformations of average signal values) are color-coded: red and green denote high expression and low expression, respectively. **(a)** leaf-preferred genes. **(b)** Ubiquitously expressed genes. **(c)** Other tissue-preferred genes.

Different naming systems have been used for the genes of the rice MAP kinase families. In this article, we used MPKKK for MAPKKK, MPKK for MAPKK, and MPK for MAPK (Additional file 1: Table S1) (Hamel et al. 2006; Reyna and Yang 2006; Rao et al. 2010). The leaf tissue is the major site of *Xoo* invasion (Kou and Wang 2013). To ascertain which MAP kinase cascade gene had high expression level in leaf tissues, a genome-wide microarray data from 28 tissues collected throughout the life cycle of two indica rice varieties in the microarray database (http://www.ncbi.nlm.nih.gov/; accession number GSE19024; Wang et al. 2010) was analyzed. In total, probes for 94 genes including probes for 70 of the 74 *MPKKK*s, 7 of the 8 *MPKK*s, and all the 17 *MPK*s were identified in the data set (Additional file 1: Table S1). The MAP kinase cascade genes in the two rice varieties showed similar expression patterns. These genes could be classified into three groups, the leaf-preferred genes, ubiquitously expressed genes, and other tissue-preferred genes (Figure 1). Only 9 genes (*MPKKK18*, *MPKKK28*, *MPKKK50*, *MPKKK51*, *MPKKK54*, *MPKKK61*, *MPKK1*, *MPKK4*, and *MPK13*), which showed higher expression level in leaf tissues than in other tissues, are in the leaf-preferred group (Figure 1a). Six of the 9 genes, except of *MPKKK18*, *MPKKK28*, and *MPKK4*, also showed leaf-preferred expression in japonica rice variety Nipponbare (Additional file 1: Figure S1; Cao et al. 2012). More than half (49) of the 94 MAP kinase cascade genes, including 34 *MPKKK*s, 3 *MPKK*s, and 12 *MPK*s, which showed similar level of expression in all the tissues examined, belongs to the ubiquitously expressed group; 38 of the 49 genes, including 25 *MPKKK*s (*1/OsEDR1*, *2*, *6*, *8*, *16*, *17*, *20*, *21*, *22*, *24*, *25*, *27*, *29*, *31*, *32*, *33*, *34*,*37*, *38*, *41*, *42*, *44*, *49*, *65*, and *72*), 2 *MPKK*s (*5* and *6*), and 11 *MPK*s (*1*, *3*, *4*, *5/MAPK5*, *6*, *7*, *9*, *10*, *11*, *12/BWMK1*, and *14*), showed high expression level in all the tissues including leaves (Figure 1b). The rest 36 genes, including 30 *MPKKK*s, 2 *MPKK*s, and 4 *MPK*s, which had higher expression level in other tissues than in leaves, are classified into the other tissue-preferred group (Figure 1c). These results suggest that approximately half of the examined MAP kinase cascade genes are highly expressed in leaf tissues when without pathogen infection.

### Analysis of the influence of bacterial infection on the expression of the three MAP kinase gene families

To explore the genes putatively involved in rice–*Xoo* interaction, two pairs of susceptible and resistant rice lines, Mudanjiang 8 versus Rb49 and IR24 versus IRBB13, were inoculated with *Xoo* strains PXO61 and PXO99, respectively. The *MR* genes, *Xa3/Xa26* carried by Rb49 and *xa13* carried by IRBB13, confer resistance to *Xoo* by different mechanisms (Sun et al. 2004; Chu et al. 2006b; Yuan et al. 2010). Rb49 is resistant to PXO61 but susceptible to PXO99, IRBB13 is resistant to PXO99 but susceptible to PXO61, and Mudanjiang 8 and IR24 are susceptible to both PXO61 and PXO99 (Sun et al. 2004; Chu et al. 2006a). The expressions of all the 74 *MPKKK*s, 8 *MPKK*s, and 17 *MPK*s were analyzed. Expressions of 61 of the 74 *MPKKK*s, 6 of the 8 *MPKK*s, and 17 *MPK*s were detected in the leaf tissue of 2 pairs of rice lines. The expression of *MPKKK59* and *MPKKK62* was only detected in the leaf tissue of one pair of rice lines (Mudanjiang 8 and Rb49). Eleven *MPKKK*s (*13*, *15*, *57*, *58*, *60*, *66*, *67*, *68*, *69*, *71*, and *73*) and 2 *MPKK*s (*10*–*1* and *10*–*3*) either did not express or had undetectable low levels of expression in the leaf tissue of all the examined rice varieties in these experimental conditions. The expression levels of all the examined genes were induced or suppressed in either the resistant reaction or the susceptible reaction, or in both reactions. To examine whether some of the observed differential expression of these genes in rice–*Xoo* interactions was resulted from circadian regulation, we checked the meta-expression data (http://ricexpro.dna.affrc.go.jp/RXP_0002/index.php; GSE36040) for circadian regulation in the field (Sato et al. 2013). Comparing with the circadian regulation data in the database and the expression patterns of the genes in resistant and susceptible rice lines with the same genetic background, the differential expression of some of the genes examined in the present study, such as 8 *MPKKK*s (*26*, *34*, *38*, *39*, *41*, *43*, *45*, and *63*), 3 *MPKK*s (*1*, *4*, and *5*), and 4 *MPK*s (*11*, *12*, *15*, and *16*), may be partially due to circadian regulation (Additional file 1: Figure S2). Because we could not exclude from the influence of *Xoo* infection on the expression of these genes, we still considered that these predicted circadian-regulated genes were also transcriptionally responsive to pathogen infection and were further analyzed.

Although all of these genes showed significantly (*P* < 0.05) induced or suppressed expression during rice–*Xoo* interaction during at least one time point examined, the levels of changed expressions of these genes were markedly different in the japonica rice lines (Mudanjiang 8 and Rb49) and the indica rice lines (IR24 and IRBB13). In the japonica background, only 16 of the 63 *MPKKK*s, 3 of the 6 *MPKK*s, and 5 of the 17 *MPK*s showed an expression change that was more than three-fold during at least one time point examined; however, in the indica background, 32 of the 61 *MPKKK*s, 6 *MPKK*s, and 10 of the 17 *MPK*s showed an expression change that was more than three-fold.

**Figure 2:**
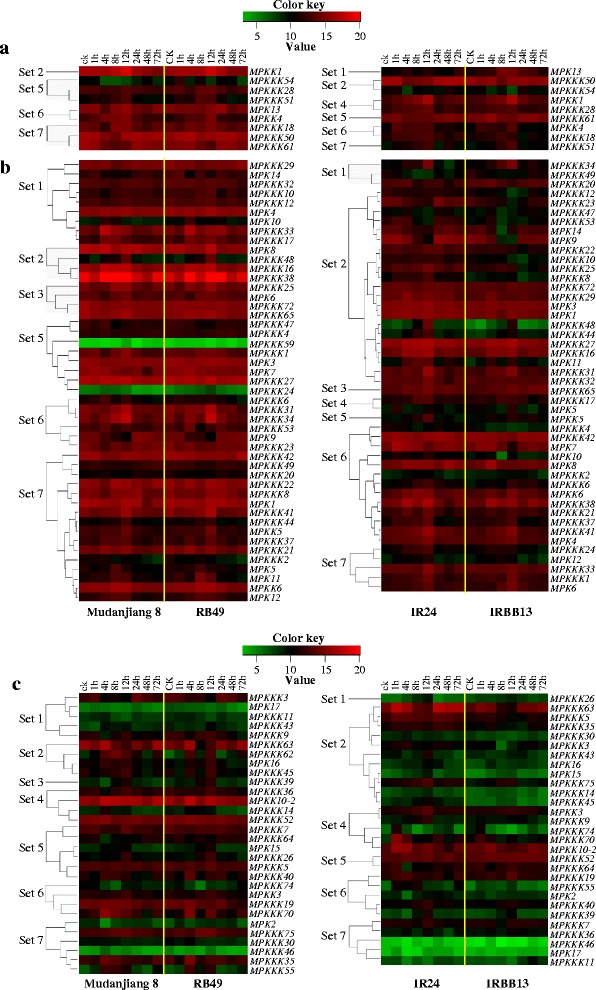
**The three anatomical/developmental grouped genes were further classified into seven sets in rice response to** ***Xoo*** **infection.** Plants were inoculated with *Xoo* strain PXO61 (Mudanjiang 8 and RB49) or PXO99 (IR24 and IRBB13) at the booting stage. ck, without *Xoo* inoculation. The expression of each gene is presented as percentage of actin gene. Expression levels (log2 transformations of average signal values) are color-coded: red and green denote high and low expression, respectively. **(a)** Leaf-preferred group. **(b)** Ubiquitously expressed group. **(c)** Other tissue-preferred group.

Comparing their transcriptional response to *Xoo* infection in each pair of susceptible and resistant rice lines, the three anatomical/developmental grouped genes, leaf-preferred, ubiquitously expressed, and other tissue-preferred, were further classified into seven sets in both of the two pairs of rice lines (Figure 2). However, not every group of each pair of rice lines contained all the seven sets of genes. In general, set 1 and set 2 genes had opposite expression patterns. The genes in set 1 were expressed at significantly higher (*P* < 0.05) levels in the resistant line than in the susceptible line, both in the absence of *Xoo* infection and during most examined time points that showed significantly different (*P* < 0.05) expression levels between susceptible and resistant lines after *Xoo* infection. Set 1 expression mostly appeared in the genes of the japonica resistant line Rb49; 10 *MPKKK*s (*3*, *9*, *10*, *11*, *12*, *17*, *29*, *32*, *33*, and *43*) and 4 *MPK*s (*4*, *10*, *14*, and *17*) in Rb49 showed this type of expression in the ubiquitously expressed and other tissue-preferred groups (Figure 1b,c). However, only 4 *MPKKK*s (19, *20*, *26*, and *34*) and one *MPK13* gene showed this type of expression in the leaf-preferred and other tissue-preferred groups of the indica resistant line IRBB13 (Figure 1a,c). The genes of set 2 were expressed at significantly lower levels (*P* < 0.05) in the resistant line than in the susceptible line both in the absence of *Xoo* infection and in most of examined time points that showed significantly different (*P* < 0.05) expression levels between susceptible and resistant lines after *Xoo* infection. Interestingly, the type of expression in set 2 mostly appeared in the genes of IRBB13. Twenty-seven *MPKKK*s (*3*, 5, *8*, *10*, *12*, *14*, *16*, *22*, *23*, *25*, *27*, *29*, *30*, *31*, *32*, *35*, *43*, *44*, *45*, *47*, *48*, *50*, *53*, *54*, *72*, *63*, and *75*) and 7 *MPK*s (*1*, *3*, *9*, *11*, *14*, *15*, and *16*) showed this type of expression in all the three groups of genes of IRBB13, whereas only 6 *MPKKK*s (*16*, *38*, *45*, *48*, *62*, and *63*), *MPKK1*, and 2 *MPKs* (*8* and *16*) genes showed this type of expression in Rb49 (Figure 2).

The set 3 and set 4 genes also showed opposite expression patterns in general. The genes in set 3 were expressed at significantly lower levels (*P* < 0.05) in the resistant line than in the susceptible line in the absence of *Xoo* infection but at significantly higher levels (*P* < 0.05) during most examined time points, which showed significantly different (*P* < 0.05) expression levels between susceptible and resistant lines, in the resistant line after *Xoo* infection. Set 3 expression was detected in 4 *MPKKK*s (*25*, *39*, *65*, and *72*) and *MPK6* in the ubiquitously expressed and other tissue-preferred groups of Rb49 rice line and only *MPKKK65* in IRBB13 rice line (Figure 2b,c). The genes of set 4 were expressed at significantly higher levels (*P* < 0.05) in the resistant line than in the susceptible line in the absence of *Xoo* infection but at significantly lower levels (*P* < 0.05) in most of examined time points, which showed significantly different (*P* < 0.05) expression levels between susceptible and resistant lines, in the resistant line after *Xoo* infection. Three *MPKKK*s (*14*, *36*, and *52*) and *MPKK10-2* in the other tissue-preferred group of Rb49 and 5 *MPKKK*s (*9*, *17*, *28*, *70*, and *74*), 3 *MPKK*s (1, *3*, and *10*–*2*), and *MPK5*/*OsMAPK5* in all the three groups of IRBB13 showed the same type of expression as set 4 genes (Figure 2).

The set 5 and set 6 genes showed similar levels of expression in resistant and susceptible rice lines in the absence of *Xoo* infection, but they had opposite expression patterns after *Xoo* infection. In most examined time points that showed significantly different (*P* < 0.05) expression levels between susceptible and resistant lines after *Xoo* infection, the genes in set 5 were expressed at significantly higher (*P* < 0.05) levels in the resistant line than in the susceptible line, whereas the genes in set 6 were expressed at significantly lower (*P* < 0.05) levels in the resistant line than in the susceptible line. Fourteen *MPKKK*s (*1*, *4*, *5*, *7*, *24*, *26*, *27*, *28*, *40*, *47*, *51*, *54*, *59*, and *64*) and 3 *MPK*s (*3*, 7, and 15) in the Rb49 rice line and 3 *MPKKK*s (*52*, *61*, and *64*) and *MPKK5* in the ubiquitously expressed and other tissue-preferred groups of IRBB13 rice line showed the same type of expression as set 5 (Figure 2). Set 6 type of expression occurred in all the three groups of genes in both rice lines Rb49 and IRBB13 (Figure 2). Eight *MPKKK*s (*6*, *19*, *23*, *31*, *34*, *53*, *70*, and *74*), 2 *MPKK*s (*3* and *4*), and 2 *MPKs* (*9* and *13*) in Rb49 and 13 *MPKKK*s (*2*, *6*, *4*, *18*, *19*, *21*, *37*, *38*, *39*, *40*, *41*, *42*, and *55*), 2 *MPKK*s (*4* and *6*), and 5 *MPK*s (*2*, *4*, *7*, *8*, and *10*) in IRBB13 showed the same type of expression as set 6 (Figure 2).

The genes in set 7 showed a similar expression pattern in resistant and susceptible rice lines, although these genes had a transcriptional response to *Xoo* infection. This type of expression also occurred in all the three groups of genes in both rice lines Rb49 and IRBB13 (Figure 2). Eighteen *MPKKK*s (*2*, *8*, *18*, *20*, *21*, *22*, *30*, 35, *37*, *41*, *42*, *44*, *46*, *49*, *50*, *55*, *61*, and *75*), 2 *MPKK*s (*5* and *6*), and 5 *MPK*s (*1*, *2*, *5/OsMAPK5*, *11*, and *12*) in the Rb49 rice line, and 8 *MPKKK*s (*1/OsEDR1*, *7*, *11*, *24*, *33*, *36*, *46*, and *51*) and 3 *MPK*s (*6*, *12*, and *17*) in the IRBB13 rice line belong to this set. These results suggest that MAP kinase cascades may be differently involved in different rice–*Xoo* interactions.

### Analysis of the co-expression of MAPKKK, MAPKK, and MAPK genes

To identify putative MAP kinase cascades in rice − *Xoo* interaction, we analyzed the co-expression of the three types of genes based on their transcriptional response to pathogen infection. Because these genes were classified into three anatomical/developmental groups and showed different expression patterns in different genetic backgrounds (Figures 1 and 2), co-expression analyses were first performed within each of the anatomical/developmental groups and further performed among the genes of all the MAP kinase families. Based on the permutation test, the optimal threshold of the Pearson correlation coefficient (PCC) values for co-expression analysis in the japonica background (rice lines Mudanjiang 8 and Rb49) and indica background (rice lines IR24 and IRBB13) were determined as 0.66 and 0.73 with a false discovery rate of 0.001, respectively (Additional file 1: Figures S3 and S4).

**Figure 3:**
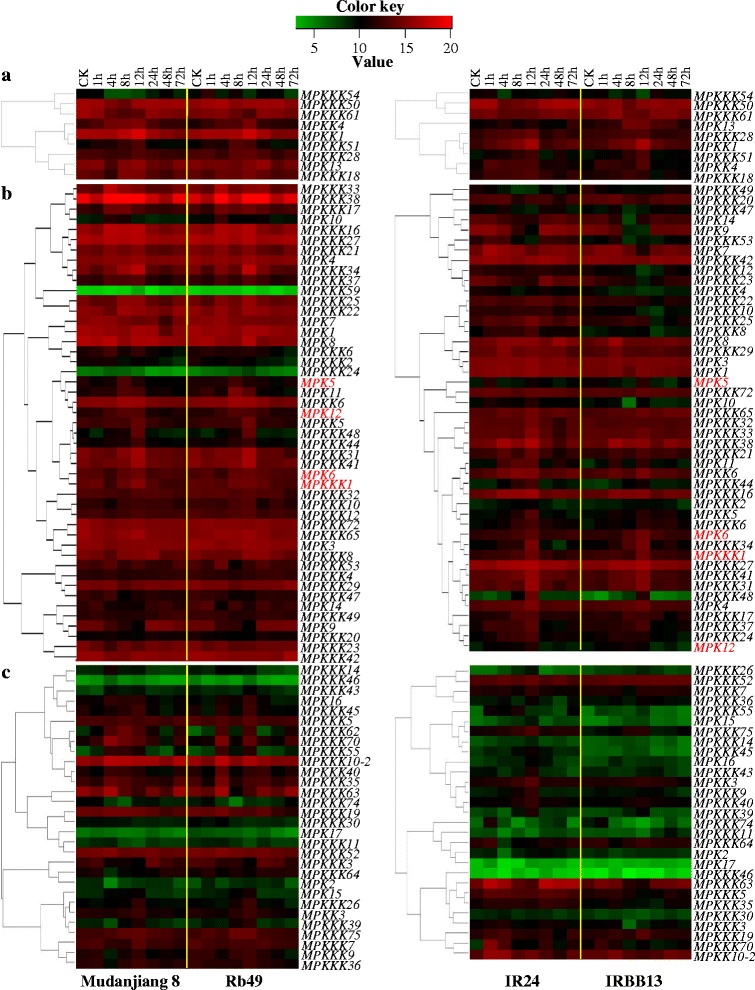
**Co-expression analysis of** ***Xoo*** **-responsive genes within each of the anatomical/developmental groups.** Plants were inoculated with *Xoo* strain PXO61 (Mudanjiang 8 and RB49) or PXO99 (IR24 and IRBB13) at the booting stage. ck, without *Xoo* inoculation. The expression of each gene is presented as percentage of actin gene. Expression levels (log2 transformations of average signal values) are color-coded: red and green denote high and low expression, respectively. The names of MAP kinase genes known to be involved in rice–*Xoo* interactions are shown in red color. **(a)** Leaf-preferred group. **(b)** Ubiquitously expressed group. **(c)** Other tissue-preferred group.

**Figure 4:**
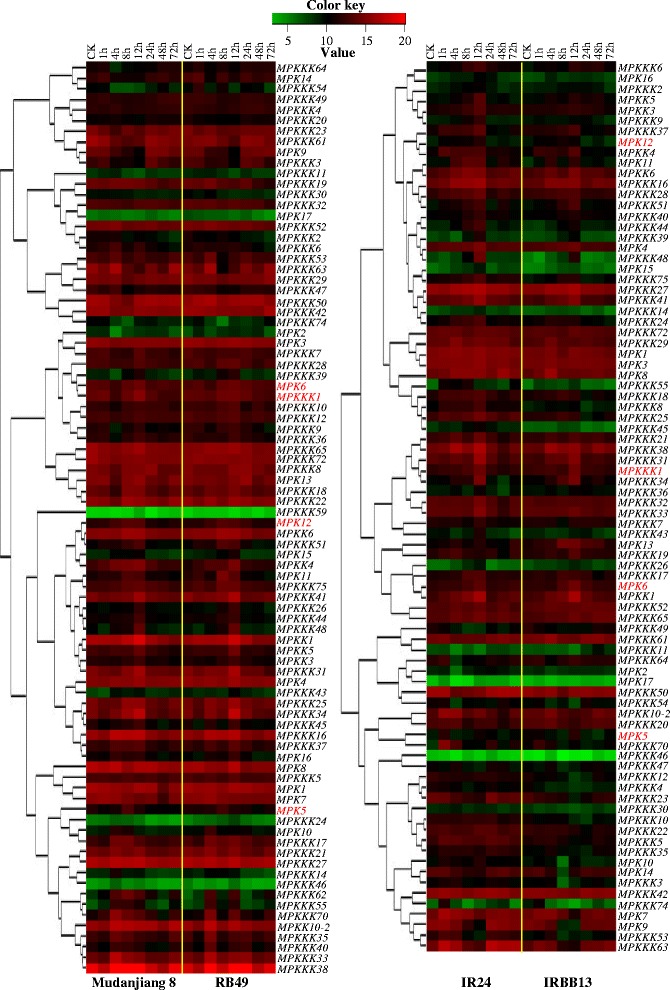
**Co-expression analysis of all** ***Xoo*** **-responsive genes.** Plants were inoculated with *Xoo* strain PXO61 (Mudanjiang 8 and RB49) or PXO99 (IR24 and IRBB13) at the booting stage. ck, without *Xoo* inoculation. The expression of each gene is presented as percentage of actin gene. Expression levels (log2 transformations of average signal values) are color-coded: red and green denote high and low expression, respectively. The names of MAP kinase genes known to be involved in rice–*Xoo* interactions are shown in red color.

According to the aboved mentioned thresholds, the *MPKKK1* and *MPK6*, which are known to be involved in rice–*Xoo* interactions (Yuan et al. 2007; Shen et al. 2010, 2011), showed co-expression in both japonica and indica backgrounds analyzed either within anatomical/developmental group and among the genes of all the MAP kinase families (Figures 3b and 4; Table 1). Only 6 *MPKK*s were detected showing pathogen-responsive expression in leaf tissues (Figure 2). Among the 6 *MPKK*s, *MPKK1*, *3*, *5*, and *6* co-expressed with both *MPKKK1* and *MPK6* with *MPKK1* and *3* having more close co-expression with *MPKK1* and *MPK6* than *MPKK5* and *6* in japonica background, and only *MPKK1* co-expressed with both *MPKKK1* and *MPK6* in the indica background (Figures 3b and 4; Table 1). The *MPK5* and *MPK12*, which are also known to regulate rice response to *Xoo* infection (Seo et al. 2011), did not co-express with *MPKKK1*; however, *MPK12* co-expressed with *MPKK1*, *3*, *4*, and *6* in japonica rice lines and with *MPKK3* and *4* in indica rice lines, *MPK5* co-expressed with *MPKK6* in japonica background (Table 1). Furthermore, *MPKK1*, *3*, *4*, *5*, and *6* each co-expressed with one or more *MPKKK*s in rice–*Xoo* interactions (Additional file 1: Tables S2 and S3).

**Table 1:** **The Pearson correlation coefficient values of co-expression between known defense-related MAP kinase genes and** ***MPKK*** **s in japonica and indica rice lines** ^**a**^

**Japonica rice lines (Mudanjiang 8 and Rb49)** ^**b**^										
	***MPKKK1***	***MPKK1***	***MPKK3***	***MPKK4***	***MPKK5***	***MPKK6***	***MPKK10-2***	***MPK5***	***MPK6***	***MPK12***
***MPKKK1***	1	0.903	0.859	0.663	0.666	0.752	−0.004	0.336	0.860	0.644
***MPKK1***	0.903	1	0.910	0.876	0.860	0.744	0.258	0.275	0.818	0.712
***MPKK3***	0.859	0.910	1	0.758	0.890	0.771	0.094	0.328	0.914	0.704
***MPKK4***	0.663	0.876	0.758	1	0.799	0.748	0.540	0.382	0.585	0.822
***MPKK5***	0.666	0.860	0.890	0.799	1	0.641	0.281	0.125	0.731	0.603
***MPKK6***	0.752	0.744	0.771	0.748	0.641	1	0.231	0.758	0.731	0.936
***MPKK10-2***	−0.004	0.258	0.094	0.540	0.281	0.231	1	0.142	−0.186	0.426
***MPK5***	0.336	0.275	0.328	0.382	0.125	0.758	0.142	1	0.375	0.747
***MPK6***	0.860	0.818	0.914	0.585	0.731	0.731	−0.186	0.375	1	0.626
***MPK12***	0.644	0.712	0.704	0.822	0.603	0.936	0.426	0.747	0.626	1
**Indica rice lines (IR24 and IRBB13)** ^**c**^										
	***MPKKK1***	***MPKK1***	***MPKK3***	***MPKK4***	***MPKK5***	***MPKK6***	***MPKK10-2***	***MPK5***	***MPK6***	***MPK12***
***MPKKK1***	1	0.979	0.755	0.670	0.774	0.700	−0.072	0.551	0.886	0.681
***MPKK1***	0.979	1	0.781	0.654	0.766	0.729	−0.106	0.488	0.930	0.697
***MPKK3***	0.755	0.781	1	0.489	0.936	0.740	−0.045	0.161	0.592	0.776
***MPKK4***	0.670	0.654	0.489	1	0.409	0.678	0.470	0.687	0.558	0.742
***MPKK5***	0.774	0.766	0.936	0.409	1	0.619	0.003	0.156	0.537	0.716
***MPKK6***	0.700	0.729	0.740	0.678	0.619	1	0.113	0.176	0.576	0.658
***MPKK10-2***	−0.072	−0.106	−0.045	0.470	0.003	0.113	1	0.249	−0.239	0.240
***MPK5***	0.551	0.488	0.161	0.687	0.156	0.176	0.249	1	0.507	0.285
***MPK6***	0.886	0.930	0.592	0.558	0.537	0.576	−0.239	0.507	1	0.531
***MPK12***	0.681	0.697	0.776	0.742	0.716	0.658	0.240	0.285	0.531	1

^a^The names of MAP kinase genes known to be involved in rice–*Xoo* interactions are shown in red color.

^b^The threshold is 0.66 with a false discovery rate of 0.001 for japonica rice lines.

^c^The threshold is 0.73 with a false discovery rate of 0.001 for indica rice lines.

All these results suggest that further examination can be conducted to determine whether the co-expressed MAP kinase cascade genes function in the same signaling pathway or in a background-specific signaling pathway in rice–pathogen interactions.

## Discussion

MAP kinase cascades are required for the regulation of various biological activities. However, the roles of most of the rice members of MAP kinase cascades are unknown. No single MAP kinase cascade has been characterized in rice physiologic processes so far. Although it is well known that MAP kinase members are mostly regulated at the post-transcriptional level by phosphorylation, genome-wide identification of MAP kinase cascades involved in a given biological activity according to kinase activity is still difficult. The present results suggest that transcriptome-based analysis of MAP kinase cascades may be a starting point to identify potential candidates in a MAP kinase cascade in rice–*Xoo* interactions..

### A relatively large numbers of MAP kinase cascade genes may be required for rice–pathogen interactions

The present results have revealed that four-fifths of the *MPKKK*s, more than two-thirds of the *MAPKK*s, and all the *MPK*s are transcriptionally responsive to *Xoo* infection. These results suggest that these genes may be directly or indirectly involved in rice–pathogen interactions, although further studies are required to determine the functions of these genes. This prediction is supported by the following evidence. First, most of the examined genes showed different expression patterns and different intensities of transcriptional response to *Xoo* infection in the Rb49 rice line carrying the dominant *MR* gene *Xa3/Xa26* and the IRBB13 rice line carrying the recessive *MR* gene *xa13*. This is consistent with the different mechanisms of *Xa3/Xa26*-mediated and *xa13*-mediated resistance to *Xoo. Xa3/Xa26* encodes an LRR-receptor kinase–like protein, and this type of protein is usually involved in PTI (Sun et al. 2004; Monaghan and Zipfel 2012). The *xa13* encodes an MtN3/saliva-type protein, and its dominant allele is a *Xoo* race–specific susceptible gene; a rice plant carrying recessive *xa13* has passive resistance to *Xoo* (Yuan et al. 2009, 2010; Zhang and Wang 2013). Second, other studies have also revealed that some rice MAP kinase cascade genes showed changed expression after pathogen invasion. For example, the data in microarray database (GEO DataSets; http://www.ncbi.nlm.nih.gov/gds/) show that the expression of 30 *MPKKK*s (*2*, *5*, *8*, *9*, *11*, *13*, *14*, *18*, *20*, *21*, *23*, *27*, *29*, *31*, *34*, *36*, *41*, *43*, *47*, *49*, *52*, *54*, *55*, *57*, *63*, *64*, *71*, *72*, *73*, and *75*), 3 *MPKK*s (*1*, *3* and *10*–*2*), and 13 *MPK*s (*1*, *2*, *3*, *5*, *6*, *7*, 8, *9*, *10*, *11*, *12*, *13*, and *17*) were induced or suppressed after rice response to the infection of *Xoo* (GSE19844, Yu et al. 2011; GSE43050, Narsai et al. 2013; GSE34192). In addition, the expression of *MPK12/BWMK1* (named *BWMK1* in He et al. 1999) was induced by *M. oryzae*. The transcripts of 9 (*2*, *4*, *5*, *7*, *8*, *12*, *13*, *15*, and *17*) of the 17 rice *MPK*s were increased more than three-fold after *M. oryzae* infection (Reyna and Yang 2006). The expression of *MPK13/OsBIMK2* (named *OSBWMK2* in Song et al. 2006) was upregulated after *M. oryzae* infection; overexpressing *MPK13/OsBIMK2* in tobacco enhanced disease resistance against tomato virus and fungal pathogens. Finally, four (*MPKKK1/OsEDR1*, *MPK5/OsMAPK5*, *MPK6*, and *MPK12/BWMK1*) of these MAP kinase cascade genes that had transcriptional responses to *Xoo* infection detected in this study have been proven to be involved in rice–*Xoo* interactions (Yuan et al. 2007; Shen et al. 2010, 2011; Seo et al. 2011). In addition, *MPKKK1/OsEDR1* and *MPK5/OsMAPK5* also regulate the rice response to *M. oryzae*, and *MPK5/OsMAPK5* also regulates the rice response to *B. glumae* (Xiong and Yang 2003; Shen et al. 2011). Furthermore, studies have revealed that rice defense signaling against pathogen and insect shares common components (Hao et al. 2011; Chen et al. 2013). A recent study has revealed that several *MPK*s (*5*, *12*, *13*, and *17*) may be involved in rice − brown planthopper interaction (Hu et al. 2011). These results suggest that MAP kinase cascade genes may play important roles in the rice responses to biotic stresses.

### More than one MAP kinase cascades might be involved in the rice response to *Xoo* infection

Signal transfer between proteins requires the co-expression of proteins. The co-expression of genes can represent, to a certain extent, the co-expression of their encoding proteins. Thus, studying the transcriptome of rice MAP kinase genes may help to identify potential MAP kinase cascades for further characterization. This hypothesis is supported by the evidence that the genes, which encoding proteins consist of known MAP kinase cascades, showed co-expression by analysis of *Arabidopsis* large scale co-expression data. For example, MEKK1–MKK4 or MKK5–MPK3 or MPK6 and MEKK1–MKK1 or MKK2–MPK4 are two MAP kinase cascades involved in *Arabidopsis*–pathogen interactions (Petersen et al. 2000; Asai et al. 2002; Ren et al. 2002; Ichimura et al. 2006; Nakagami et al. 2006; Suarez-Rodriguez et al. 2007; Qiu et al. 2008; Kong et al. 2012; Zhang et al. 2012). The data collected in The Arabidopsis Information Resource (http://www.arabidopsis.org; Obayashi et al. 2009) show that *MEKK1* co-expressed with *MKK4*, *MKK4* co-expressed with *MPK3*, *MKK1* co-expressed with *MKK2*, and *MKK2* co-expressed with *MPK4*.

**Figure 5:**
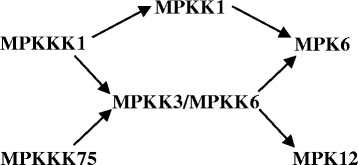
**A proposed MAP kinase cascade model consisting of seven members in rice–** ***Xoo*** **interaction.** The model is proposed based on co-expression analysis of MAP kinase genes in rice response to *Xoo* infection.

A signal transfer in a MAP kinase cascade usually flows from a MAPKKK to a MAPKK and, in turn, to a MAPK via phosphorylation. To indentify the candidates of MAP kinase cascades involved in rice–*Xoo* interactions, we focused on the co-expression of the four known defense-related MAP kinase genes (*MPKKK1/OsEDR1*, *MPK5/OsMAPK5*, *MPK6*, and *MPK12/BWMK1*) with six *MPKK*s that were transcriptionally response to *Xoo* infection in the present study (Table 1). Based on this analysis, MPKKK1/OsEDR1–MPKK1–MPK6 could be a candidate of MAP kinase cascade involved in rice response to *Xoo* infection in both japonica and indica rice lines in the present experimental conditions (Figure 5). The second candidate cascade is MPKKK1/OsEDR1–MPKK3 or MPKK6–MPK6, which may only occur in the japonica rice lines but not the indica rice lines (Figure 5). The third candidate cascade could be MPKKK1/OsEDR1 or MPKKK75–MPKK3 or MPKK6–MPK12/BWMK1 in both the japonica and indica rice lines (Figure 5). This inference is supported by the following evidence. First, three proteins (*MPKKK1/OsEDR1*, *MPK6*, and *MPK12/BWMK1*) of the predicted cascades have been proven to regulate the rice response to *Xoo*; both MPKKK1/OsEDR1 and MPK6, which are predicted in the same cascade, negatively regulate the rice resistance to *Xoo* in the same rice line (Yuan et al. 2007; Shen et al. 2010, 2011). Second, some MAP kinase cascade proteins have been detected to interact with each other physically. For example, MPKK1 (named MEK2 previously) and MPKK6 (named MEK6 and MKK6 previously) interact with MPK6 in yeast, tobacco, and rice cells (Singh et al. 2012; Wankhede et al. 2013). Third, previous studies have also reported that *MPKKK1/OsEDR1*, *MPKKK75*, *MPKK1*, *MPKK3*, *MPKK6*, *MPK6*, and *MPK12/BWMK1* were transcriptional response to *Xoo* infection (Yuan et al. 2007; Shen et al. 2011; Yu et al. 2011; Narsai et al. 2013). Fourth, a MAP kinase cascade frequently contains redundant components. For example, two MAP kinase cascade, such as AtMEKK1–AtMKK4 or AtMKK5–AtMPK3 or AtMPK6, and AtMEKK1–AtMKK1 or AtMKK2–AtMPK4 functions in *Arabidopsis* innate immunity (Asai et al. 2002). Another *Arabidopsis* MAP kinase cascade also consisting of redundant components, such as AtEDR1–AtMKK4 or AtMKK5–AtMPK3 or AtMPK6, is also involved in innate immunity (Zhao et al. 2014). Finally, *Arabidopsis* and rice orthologs frequently have conserved biological functions. Rice MPKKK1/OsEDR1, MPKK1, and MPK6 are *Arabidopsis* orthologs of defense-related AtEDR1, AtMKK1/AtMKK2, and AtMPK4, respectively (Hamel et al. 2006; Yuan et al. 2007; Ke et al. 2014). Thus, the present results provide candidate MAP kinase cascades for further studies of the defense signaling in rice–*Xoo* interactions.

A previous study has suggested another MAP kinase cascade, MPKKK55 or MPKKK57–MPKK4–MPK5/OsMAPK5, may regulate rice response to the invasion of pathogen and insect by analyzing transcriptome data (Jung et al. 2010). The present results showed that *MPK5/OsMAPK5* did not co-express with any of the *MPKK*s that showed changed expression after *Xoo* infection (Table 1). All these results suggest that multiple MAP kinase cascades may be involved in rice–pathogen interactions. However, these cascades may be preferentially involved in different types of host–pathogen interaction. This inference is supported by the present evidence that a large number of MAP kinase genes showed different expression patterns based our 7 sets of classification between japonica (Rb49) and indica (IRBB13) resistant rice lines after *Xoo* infection (Figure 2). Rice lines Rb49 and IRBB13 carries *MR* genes *Xa3/Xa26* and *xa13*, which confer resistance against *Xoo* by different mechanisms, respectively (Sun et al. 2004; Chu et al. 2006b; Yuan et al. 2010). Thus, further studies may be required to examine the MAP kinase cascade candidates in different rice lines that carrying different types of *MR* genes or without carrying *MR* genes.

Studies have also revealed that a MAPK may phosphorylate a MAPKKK during negative feedback regulation of the MAP kinase cascade. For example, mammalian Raf-1 (a MAPKKK) can be negatively regulated by the feedback phosphorylation of ERK2 (a MAPK) both *in vitro* and *in vivo* (Dougherty et al. 2005). The kinase activity of B-Raf (a MAPKKK) is negatively regulated by ERK2 at mitosis in *Xenopus* eggs (Borysov et al. 2006; McKay et al. 2009). The present results have revealed that a relatively large number of *MPKKK* genes were co-expressed with *MPK* genes in rice–*Xoo* interactions (Figures 3 and 4; Table 1), although there is no report indicating that a rice MPK can phosphorylate a MPKKK. Thus, further studies are needed to examine whether these MPKs regulate MAP kinase cascades by feedback phosphorylation of MPKKKs.

## Conclusions

The present results suggest that a relatively large number of MAP kinase cascade genes may be required for rice–pathogen interactions, although only four of these genes have been reported to be positively or negatively involved in rice disease resistance (Yuan et al. 2007; Shen et al. 2010, 2011; Seo et al. 2011). The encoding proteins of these genes may form multiple MAP kinase cascades in the rice response to *Xoo* infection in different genetic backgrounds. These results will facilitate further functional and biochemical characterization of these protein kinases and the exploration of the roles of MAP kinase cascades in rice disease resistance.

## Methods

### Materials

Two pairs of susceptible and resistant rice lines, each with the same genetic background, were used for the analysis. The first pair of rice lines comprised Rb49 and IR24. Transgenic line Rb49 carries a *MR* gene *Xa3/Xa26* driven by its native promoter and encodes a plasma membrane–localized leucine-rich repeat receptor kinase–like protein conferring race-specific resistance to *Xoo* (Sun et al. 2004; Xiang et al. 2006). The wild-type Mudanjiang 8 is a japonica rice variety (*Oryza sativa* ssp. *japonica*) and is susceptible to *Xoo*. The second pair comprised IRBB13 and IR24 near-isogenic indica rice (*O. sativa* ssp. *indica*) lines. IRBB13 carries a recessive *MR* gene *xa13*, which confers race-specific resistance to *Xoo*, with the genetic background of IR24; however, IR24 is susceptible to *Xoo* (Chu et al. 2006a). The *xa13* confers resistance by nonresponse to pathogen-induced expression, which leads to the maintenance of a concentration of copper that is toxic to *Xoo* in the xylem vessels (Chu et al. 2006b; Yuan et al. 2010).

### *Xoo* inoculation

Plants were inoculated with Philippine *Xoo* strain PXO61 or PXO99 by the leaf-clipping method at the booting (panicle development) stage (Chen et al. 2002). *Xoo* infection was performed at 7:00. Sample from control (ck) was collected immediately before inoculation of *Xoo*. The 3-cm leaf fragments near the bacterial infection sites were collected for RNA isolation.

### Quantitative reverse-transcription polymerase chain reaction

Total RNA was used for gene expression analyses by quantitative reverse-transcription polymerase chain reaction (qRT-PCR) (Qiu et al. 2007). PCR primers are listed in Additional file 1: Table S4. For the data presented as heatmap, the expression of the actin gene was used as the internal control; the expression of each gene was presented using percentage of actin gene. For the data presented as bar figure, the expression level of the actin gene was first used to standardize the RNA sample for each qRT-PCR, and then the expression level relative to control was calculated.

### Genome-wide data collection and gene co-expression analysis

Microarray data for indica rice varieties Minghui 63 and Zhenshan 97 were collected from a microarray database (http://www.ncbi.nlm.nih.gov/; accession number GSE19024), which covers 28 tissues and organs representing the entire life cycle of rice (Wang et al. 2010). The tissue-specific and development-specific expression data for MAP kinase genes were downloaded from the Collection of Rice Expression Profiles database (http://crep.ncpgr.cn/crep-cgi/home.pl). The expression of the genes was presented by a hierarchical cluster displaying based on Pearson correlation coefficient (PCC) values.

The co-expression between MAPKKK, MAPKK, and MAPK genes was analyzed by calculating the PCC values between all pairs of genes (Carter et al. 2004). We used the permutation test to determine the optimal threshold of the PCC for gene co-expression analysis (Ouyang et al. 2012). The distribution of PCCs between all pairwised comparison of MAP kinase genes were plotted before and after independent random permutation. The optimal threshold of the PCC was determined with a false discovery rate of 0.001.

### Statistical analysis

Differences between samples were analyzed for statistical significance by the method of the pairwise *t*-test in Microsoft Excel (Microsoft, Redmond, WA).

## Additional file

Additional file 1: Figure S1. Expression profiles of mitogen-activated protein kinase genes in different rice tissues and organs in rice variety Nipponbare. Data were obtained from a microarray database (http://www.ricearray.org/expression/meta_analysis.shtml; accession number GSE21396; Cao et al. 2012). Expression levels (log2 transformations of average signal values) are color-coded: yellow and blue denote high and low expression, respectively. Leaf-related tissues/organs are labeled with a red triangle. Leaf-preferred genes are named with red color. **Figure S2.** The expression of some mitogen-activated protein kinase genes which might be partially affected by circadian regulation. Plants were inoculated with *Xoo* strain PXO61 (a) or PXO99 (b) at the booting stage. ck, without *Xoo* inoculation. Data are means (three replicates) ± standard deviations. The letters “a” and “b” indicate statistically significant differences between ck and infected plants of the same rice line at *P* < 0.01 and *P* < 0.05, respectively. Asterisks indicate statistically significant differences between resistant and susceptible plants subjected to the same treatment at ***P* < 0.01 and **P* < 0.05. **Figure S3.** The distribution of Pearson correlation coefficient (PCC) values based on the expression of mitogen-activated protein kinase genes in japonica rice lines Mudanjiang 8 and Rb49 after infection of *Xoo* strain PXO61. The optimal threshold of the PCC was determined as 0.66 with a false discovery rate of 0.001. **Figure S4.** The distribution of Pearson correlation coefficient (PCC) values based on the expression of mitogen-activated protein kinase genes in indica rice lines IR24 and IRBB13 after infection of *Xoo* strain PXO99. The optimal threshold of the PCC was determined as 0.73 with a false discovery rate of 0.001. **Table S1.** Mitogen-activated protein kinase cascade genes in the rice genome. **Table S2.** The Pearson correlation coefficient values of co-expression between *MPKKK*s and *MPKK*s in japonica rice lines Mudanjiang 8 and Rb49. **Table S3.** The Pearson correlation coefficient values of co-expression between *MPKKK*s and *MPKK*s in indica rice lines IR24 and IRBB13. **Table S4.** Polymerase chain reaction (PCR) primers used for quantitative reverse-transcription PCR assays.

## Abbreviations

ETI: Effector-triggered immunity
MAP: Mitogen-activated protein
MAPK: Mitogen-activated protein kinase
MAPKK: Mitogen-activated protein kinase kinase
MAPKKK: Mitogen-activated protein kinase kinase kinase
NB-LRR: Nucleotide-binding leucine-rich repeat
PCR: Polymerase chain reaction
PTI: Pathogen-associated molecular pattern–triggered immunity
qRT: Quantitative reverse-transcription
*Xoo*: *Xanthomonas oryzae* pv. *oryzae*
