# Supplementary material for: Transcriptome-based analysis of mitogen-activated protein kinase cascades in the rice response to Xanthomonas oryzae infection
Source: Rice (N Y). 2015 Jan 27;8:4. doi: 10.1186/s12284-014-0038-x (PMC4311651; doi:10.1186/s12284-014-0038-x)
Supplement: Additional file 1: Figure S1. — Expression profiles of mitogen-activated protein kinase genes in different rice tissues and organs in rice variety Nipponbare. Data were obtained from a microarray database (http://www.ricearray.org/expression/meta_analysis.shtml; accession number GSE21396; Cao et al. 2012). Expression levels (log2 transformations of average signal values) are color-coded: yellow and blue denote high and low expression, respectively. Leaf-related tissues/organs are labeled with a red triangle. Leaf-preferred genes are named with red color. Figure S2. The expression of some mitogen-activated protein kinase genes which might be partially affected by circadian regulation. Plants were inoculated with Xoo strain PXO61 (a) or PXO99 (b) at the booting stage. ck, without Xoo inoculation. Data are means (three replicates) ± standard deviations. The letters “a” and “b” indicate statistically significant differences between ck and infected plants of the same rice line at P < 0.01 and P < 0.05, respectively. Asterisks indicate statistically significant differences between resistant and susceptible plants subjected to the same treatment at **P < 0.01 and *P < 0.05. Figure S3. The distribution of Pearson correlation coefficient (PCC) values based on the expression of mitogen-activated protein kinase genes in japonica rice lines Mudanjiang 8 and Rb49 after infection of Xoo strain PXO61. The optimal threshold of the PCC was determined as 0.66 with a false discovery rate of 0.001. Figure S4. The distribution of Pearson correlation coefficient (PCC) values based on the expression of mitogen-activated protein kinase genes in indica rice lines IR24 and IRBB13 after infection of Xoo strain PXO99. The optimal threshold of the PCC was determined as 0.73 with a false discovery rate of 0.001. Table S1. Mitogen-activated protein kinase cascade genes in the rice genome. Table S2. The Pearson correlation coefficient values of co-expression between MPKKKs and MPKKs in japonica rice lines Mudanjiang 8 and Rb49. [file 12284_2014_38_MOESM1_ESM.pdf]

Transcriptome-based analysis of mitogen-activated protein kinase cascades in the rice response to *Xanthomonas oryzae* infection

Zeyu Yang, Haigang Ma, Hanming Hong, Wen Yao, Weibo Xie, Jinghua Xiao, Xianghua Li, Shiping Wang

Additional file 1

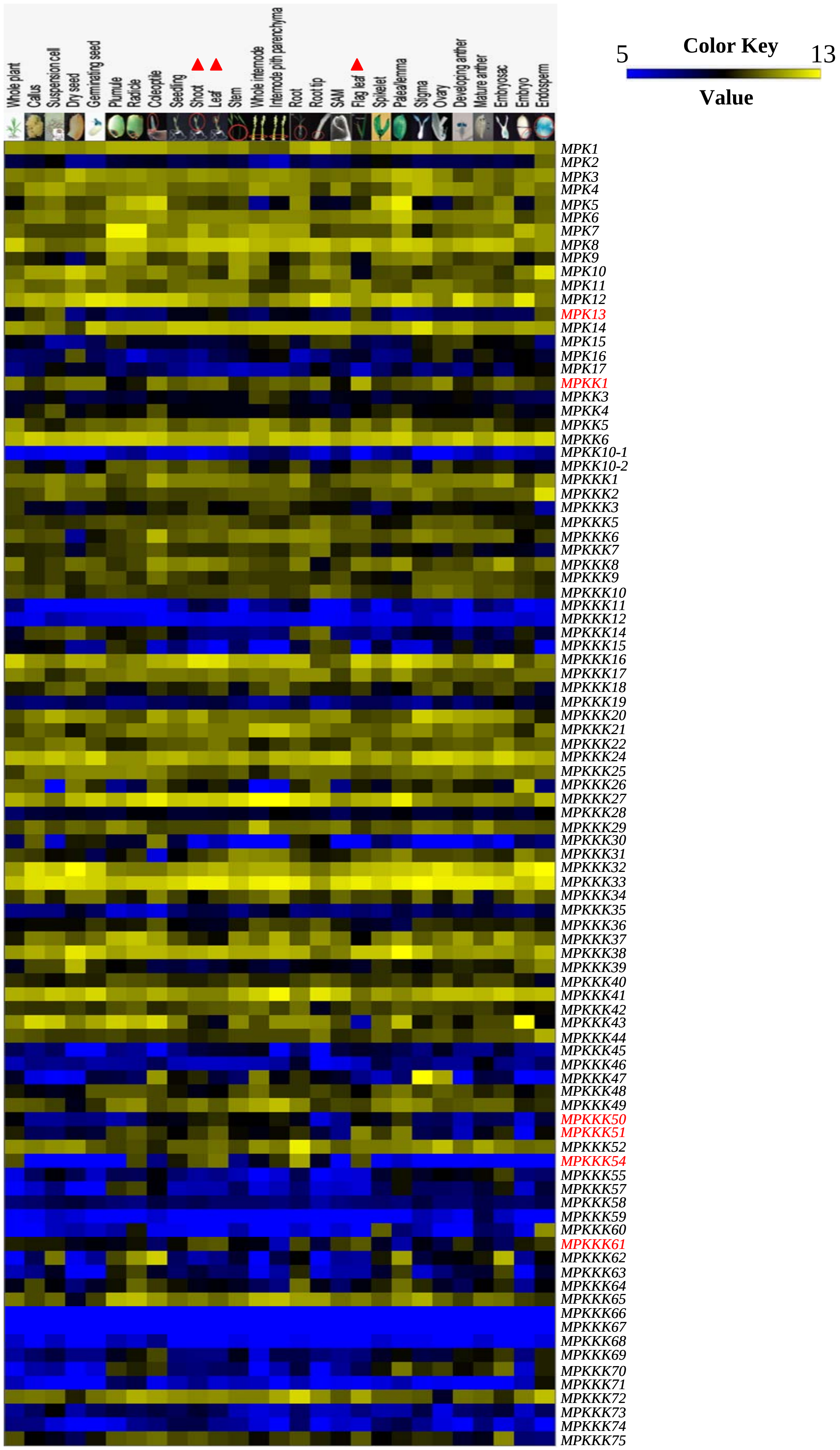

Figure S1. Expression profiles of mitogen-activated protein kinase genes in different rice tissues and organs in rice variety Nipponbare. Data were obtained from a microarray database (http://www.ricearray.org/expression/meta_analysis.shtml; accession number GSE21396; Cao et al. 2012). Expression levels (log2 transformations of average signal values) are color-coded: yellow and blue denote high and low expression, respectively. Leaf-related tissues/organs are labeled with a red triangle. Leaf-preferred genes are named with red color.

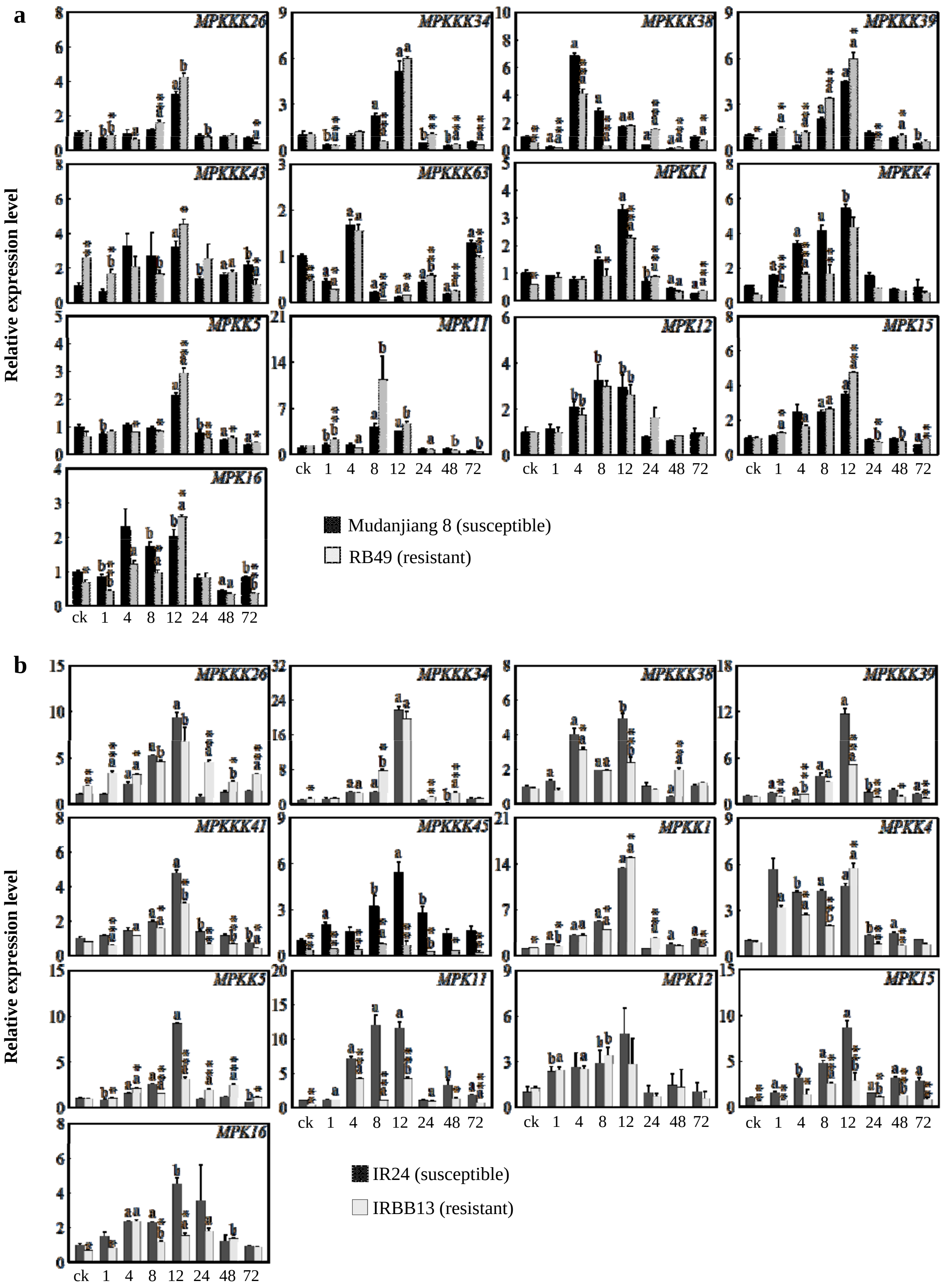

Figure S2. The expression of some mitogen-activated protein kinase genes which might be partially affected by circadian regulation. Plants were inoculated with *Xoo* strain PXO61 (**a**) or PXO99 (**b**) at the booting stage. ck, without *Xoo* inoculation. Data are means (three replicates) ± standard deviations. The letters "a" and "b" indicate statistically significant differences between ck and infected plants of the same rice line at $P < 0.01$ and $P < 0.05$, respectively. Asterisks indicate statistically significant differences between resistant and susceptible plants subjected to the same treatment at $^{**}P < 0.01$ and $^{*}P < 0.05$.

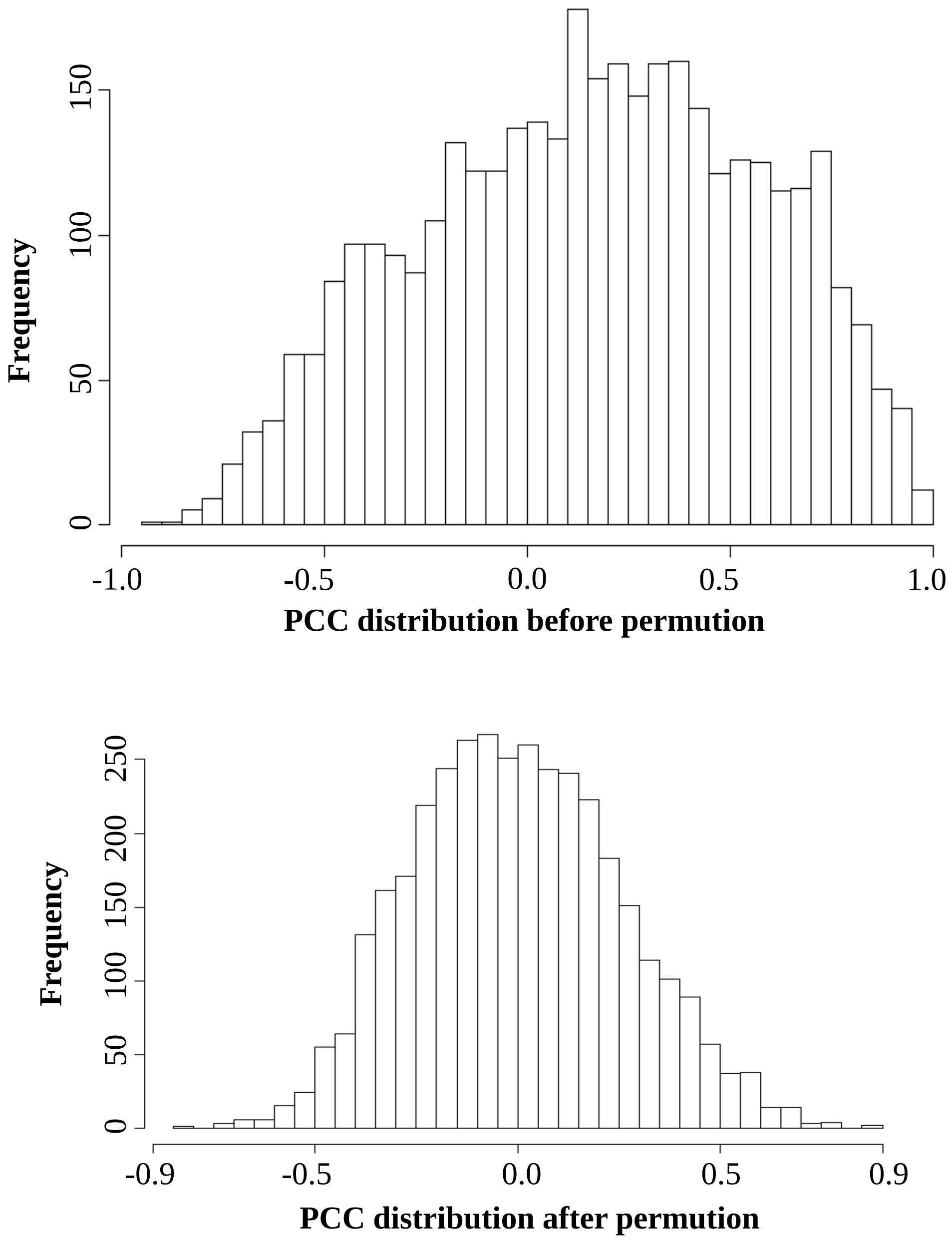

Figure S3. The distribution of Pearson correlation coefficient (PCC) values based on the expression of mitogen-activated protein kinase genes in japonica rice lines Mudanjiang 8 and Rb49 after infection of *Xoo* strain PXO61. The optimal threshold of the PCC was determined as 0.66 with a false discovery rate of 0.001.

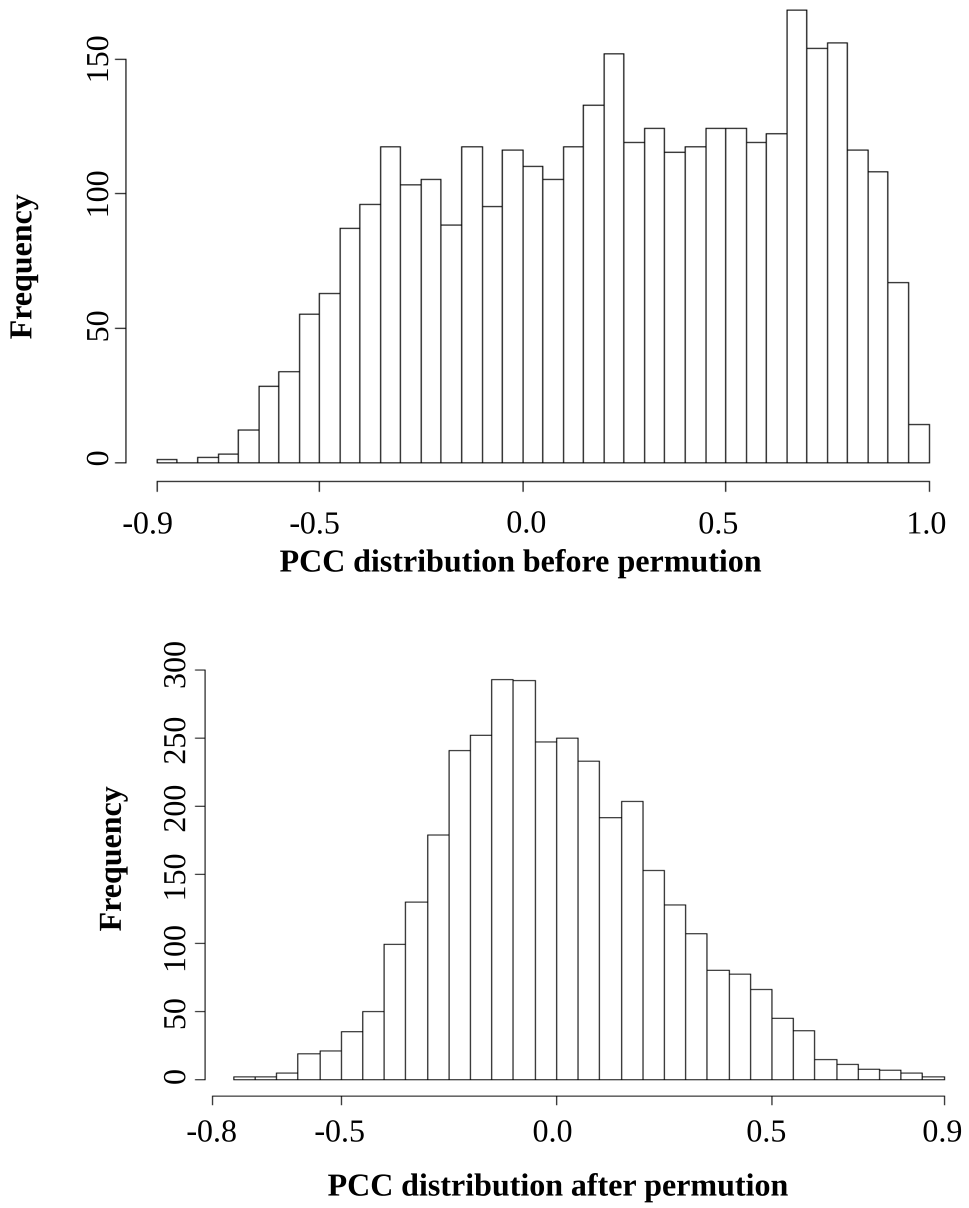

Figure S4. The distribution of Pearson correlation coefficient (PCC) values based on the expression of mitogen-activated protein kinase genes in indica rice lines IR24 and IRBB13 after infection of *Xoo* strain PXO99. The optimal threshold of the PCC was determined as 0.73 with a false discovery rate of 0.001.

Table S1. Mitogen-activated protein kinase cascade genes in the rice genome

Gene name (GenBank accession number)[a]	RGAP code[b]	cDNA[c]	Amino acids	Gene expression probeset ID[d]
MPK1	Os06g06090	AB183398	398	Os.19011.1.S1_at
MPK2	Os08g06060	BAC99508	392	OsAffx.29059.1.S1_at
MPK3	Os02g05480	AAG40581	370	Os.12683.1.S1_at
MPK4	Os06g48590	CAB61889	369	Os.4602.2.S1_a_at
MPK5/MAPK5[e] (AF479883/AF479884)	Os03g17700	AF479883	369	Os.406.1.S1_a_at
MPK6[f] (EF174189)	Os10g38950	NM_197522	386	Os.15044.1.S1_at
MPK7	Os05g49140	AK099472	599	Os.11617.1.S1_at
MPK8	Os01g47530	AJ512643	569	Os.14999.2.S1_at
MPK9	Os05g50560	AAT44204	721	Os.52787.1.S1_at
MPK10	Os01g43910	NM_192924	651	Os.26152.1.S1_at
MPK11	Os06g26340	BAD69155	570	Os.27337.1.S1_a_at
MPK12/BWMK1[g]	Os06g49430	AF177392	506	Os.7913.1.S1_a_at
MPK13	Os02g04230	AY524973	506	Os.2356.1.S1_a_at
MPK14	Os05g05160	AAS98446	542	Os.26547.1.S1_at
MPK15	Os11g17080	ABA92667	498	Os.52734.1.S1_at
MPK16	Os01g45620	NM_192298	568	Os.34984.1.A1_at
MPK17	Os05g50120	AAT39148	581	Os.34647.1.S1_at
MPKK1/MEK2[h]	Os06g05520	AK111598	352	Os.8016.1.S1_a_at
MPKK3	Os06g27890	AK103805	523	Os.49380.1.S1_s_at
MPKK4	Os02g54600	AK120525	369	Os.9331.1.S1_at
MPKK5	Os06g09180	AK112016	342	Os.49853.1.S1_a_at

MPKK6/MEK1/MKK6[i]	Os01g32660	AK059461	355	Os.2619.1.S1_at
MPKK10-1	Os02g46760		340	OsAffx.12527.1.S1_at
MPKK10-2	Os03g12390	AK104423	339	Os.16872.1.S1_at
MPKK10-3	Os03g50550		345	Not found
MPKKK1/OsEDR1[j] (KM103910)	Os03g06410	AK111595	1017	Os.2622.1.S1_at
MPKKK2	Os10g29540	AK121718	972	Os.46677.1.S1_at
MPKKK3	Os02g32610	CT835420	781	Os.8653.1.S1_at
MPKKK4	Os02g12810	AK243248	864	Not found
MPKKK5	Os12g37570	AK100149	758	Os.57425.1.S1_x_at
MPKKK6	Os02g50970	AK102767	1111	Os.5102.2.S1_at
MPKKK7	Os06g12590	AK105681	1078	Os.14975.1.S1_at
MPKKK8	Os11g10100	AK120290	653	Os.2444.1.S1_at
MPKKK9	Os02g44642	AK073040	894	Os.49462.1.S1_at
MPKKK10	Os04g47240	AK065413	894	Os.11767.1.S1_at
MPKKK11	Os07g02780	AK069889	753	Os.34851.1.S1_at
MPKKK12	Os09g39320	AK318529	1220	OsAffx.30205.1.S1_at
MPKKK13	Os09g21510	AK287525	848	Not found
MPKKK14	Os04g52140	AK120898	778	Os.14963.1.S1_at
MPKKK15	Os08g32600	AK103087	690	Os.32734.1.S1_at
MPKKK16	Os04g35700	AK061622	708	Os.27038.1.S1_at
MPKKK17	Os09g37230	AK072690	603	Os.18663.1.S1_at
MPKKK18	Os03g55560	AK106496	777	Os.37832.1.S1_at
MPKKK19	Os02g35010	AK287889	690	OsAffx.12356.1.S1_at
MPKKK20	Os07g38530	AK100426	704	Os.2221.1.S1_a_at
MPKKK21	Os07g25680	AK241123	1219	OsAffx.28586.1.S1_at
MPKKK22	Os03g49640	AK100023	654	Os.14595.1.S1_at

MPKKK23	Os12g40279		4262	Not found
MPKKK24	Os04g56530	AK099839	1357	Os.11626.1.S1_at
MPKKK25	Os02g38080	AK067412	352	Os.18535.1.S1_at
MPKKK26	Os07g29330	AK066198	439	Os.12688.1.S1_at
MPKKK27	Os03g43760	AK243690	379	Os.12681.1.A1_at
MPKKK28	Os03g15570	AK242766	597	Os.15814.1.S1_at
MPKKK29	Os02g45130	AK073845	612	Os.18183.1.S1_at
MPKKK30	Os02g02780	AK060220	583	Os.26226.1.S1_at
MPKKK31	Os01g45380	AK108130	388	Os.30916.1.S1_at
MPKKK32	Os08g12750	AK099003	418	Os.12331.1.S1_at
MPKKK33	Os02g07790	AK101327	421	Os.8323.1.S1_a_at
MPKKK34	Os05g50190	AK107217	381	Os.22907.1.S1_at
MPKKK35	Os02g54510	AK067937	1083	Os.22443.1.S1_at
MPKKK36/56	Os05g01780	AK070061/ AY336987	621	Os.49298.1.S1_at
MPKKK37	Os04g51950	AK111698	422	Os.47874.1.S1_at
MPKKK38	Os06g45300	AK112024	428	Os.11751.1.S1_a_at
MPKKK39	Os06g08280	AK067771	1273	Os.37872.1.S1_at
MPKKK40	Os01g48330	AK070808	801	Os.15379.1.S1_a_at
MPKKK41	Os06g43840	AK243413	1112	Os.17305.1.A1_s_at
MPKKK42	Os03g60150	CT830832	383	Os.9250.1.S1_at
MPKKK43	Os06g50920	AK073747	564	Os.53650.1.S1_at
MPKKK44	Os02g14530	AK111618	790	Os.8740.1.S1_at
MPKKK45	Os06g43030	AK119586	398	Os.50788.1.A1_at
MPKKK46	Os11g06140	AK070490	439	Os.53021.1.S1_at
MPKKK47	Os07g08750	AK060552	601	Os.9515.1.S1_at
MPKKK48	Os01g01740	AK059460	376	Os.23182.1.S1_at

MPKKK49	Os05g44290	AK068419	604	Os.12105.1.S1_a_at
MPKKK50	Os12g02250	AK067447	619	Os.28385.3.S1_at
MPKKK51	Os01g54350	AK102467	637	Os.2128.1.S1_at
MPKKK52	Os12g06490	AK062812	418	Os.8100.1.S1_at
MPKKK53	Os11g02305	AK071924	622	Not found
MPKKK54	Os03g28300	CT828499	859	Os.14381.1.S1_at
MPKKK55	Os01g50400	AK107168	418	Os.27170.1.S1_at
MPKKK57	Os05g46750	AK109702	591	Os.56257.1.S1_at
MPKKK58	Os03g39150		351	OsAffx.25429.1.S1_at
MPKKK59	Os12g41260	AK109696	400	Os.56254.1.S1_at
MPKKK60	Os03g53410		407	OsAffx.25701.1.S1_at
MPKKK61	Os01g10450	AK069537	563	Os.7304.1.S1_at
MPKKK62	Os01g50420	AK105196	541	Os.32366.1.S1_at
MPKKK63	Os01g50370	AK058518	484	Os.5940.1.S1_at
MPKKK64	Os07g39520	AK072014	327	Os.16320.1.S1_at
MPKKK65	Os07g43900	AK241519	321	OsAffx.16705.1.S1_at
MPKKK66	Os10g04010		525	OsAffx.30269.2.S1_at
MPKKK67	Os10g04000		526	OsAffx.30269.1.S1_at
MPKKK68	Os12g30570		384	OsAffx.19903.1.S1_at
MPKKK69	Os05g46760	AK105946	441	Os.6085.1.S1_at
MPKKK70	Os01g50410	AK071585	451	Os.5044.1.S1_at
MPKKK71	Os02g21700		413	OsAffx.12151.1.S1_at
MPKKK72	Os01g54480	AK104674	468	Os.8233.1.S1_at
MPKKK73	Os03g18170	AK108043	511	Os.55415.1.S1_at
MPKKK74	Os01g66860	AK070097	500	Os.37969.2.S1_at
MPKKK75	Os02g39560	AK071922	502	Os.53306.1.S1_at

[a]The names of MPKKKs, MPKKs, and MPKs are used according to Rao et al. (2010), Hamel et al. (2006), and Reyna and Yang (2006),

respectively.

[b]RGAP (Rice Genome Annotation Project: http://rice.plantbiology.msu.edu/) locus identifier.

[c]GenBank accession number of the full-length cDNA in the KOME database (http://cdna01.dna.affrc.go.jp/cDNA/).

[d]Probeset ID of MAPK cascade genes obtained from CREP (http://crep.ncpgr.cn/crep-cgi/home.pl).

[e]Xiong L, Yang Y (2003) Disease resistance and abiotic stress tolerance in rice are inversely modulated by an abscisic acid–inducible mitogen-activated protein kinase. Plant Cell 15:745–759

[f]Shen X, Yuan B, Liu H, Li X, Xu C, Wang S (2010) Opposite functions of a rice mitogen-activated protein kinase during the process of resistance against *Xanthomonas oryzae*. Plant J 64:86–99

[g]He C, Fong SH, Yang D, Wang GL (1999) BWMK1, a novel MAP kinase induced by fungal infection and mechanical wounding in rice. Mol Plant-Microbe Interact 12:1064–1073

[h, i]Singh R, Lee M-O, Lee, J-E, Choi J, Park JH, Kim EH, Yoo RH, Cha, J-I, Jeon J-S, Rakwal R, Agrawal GK, Moon JS, Jwa N-S (2012) Rice mitogen activated protein kinase interactome analysis using the yeast two-hybrid system. Plant Physiol 160:477–487

[i]Wankhede DP, Misra M, Singh P, Sinha AK (2013) Rice motigen activated protein kinase kinase and mitogen activated protein kinase interaction network revealed by *in-silico* docking and yeast two-hybrid approaches. PLOS One 8:e65011

[j]Shen X, Liu H, Yuan B, Li X, Xu C, Wang S (2011) OsEDR1 negatively regulates rice bacterial resistance via activation of ethylene biosynthesis. Plant Cell Environ 34:179–191

Table S2. The Pearson correlation coefficient values of co-expression between *MPKKK*s and *MPKK*s in japonica rice lines Mudanjiang 8 and Rb49[a]

	MPKKK																						
	1	*9*	*16*	*18*	*24*	*25*	*26*	*27*	*28*	*31*	*34*	*36*	*37*	*39*	*41*	*43*	*44*	*45*	*48*	*51*	*62*	*65*	*75*
MPKK1	0.90		0.68	0.78		0.76	0.85			0.88	0.90	0.72	0.77	0.83	0.77		0.94	0.87	0.90	0.77		0.79	0.70
MPKK3	0.86	0.72		0.74		0.75	0.92		0.72	0.93	0.90	0.75	0.71	0.89	0.87		0.90	0.81	0.90	0.81		0.78	0.78
MPKK4	0.66		0.87	0.70		0.75	0.75	0.66		0.75	0.83		0.87	0.72		0.69	0.84	0.80	0.81	0.71	0.76		0.67
MPKK5	0.67		0.67		0.75	0.76	0.96			0.94	0.95	0.67	0.77	0.90	0.93	0.72	0.88	0.85	0.96	0.71		0.71	0.72
MPKK6	0.75		0.71	0.76	0.71	0.69				0.68			0.67	0.77			0.75			0.91		0.72	0.88

[a]The threshold is 0.66 with a false discovery rate of 0.001 for japonica rice lines.

Table S3. The Pearson correlation coefficient values of co-expression between *MPKKK*s and *MPKK*s in indica rice lines IR24 and IRBB13[a]

	MPKKK																												
	1	***2***	***6***	***8***	***9***	***14***	***16***	***17***	***21***	***24***	***25***	***26***	***27***	***28***	***31***	***32***	***33***	***34***	***37***	***38***	***39***	***40***	***41***	***43***	***44***	***45***	***48***	***51***	***75***
MPKK1	**0.98**		**0.76**									**0.86**	**0.81**	**0.77**	**0.89**		**0.77**	**0.97**	**0.84**		**0.85**	**0.83**	**0.90**		**0.75**		**0.75**	**0.95**	
MPKK3	**0.75**	**0.84**	**0.88**	**0.77**	**0.97**	**0.83**	**0.79**	**0.85**	**0.79**	**0.90**	**0.74**	**0.85**	**0.79**	**0.89**	**0.86**	**0.77**	**0.84**	**0.79**	**0.84**		**0.95**	**0.96**	**0.91**	**0.85**	**0.96**	**0.79**	**0.92**		**0.90**
MPKK4																			**0.81**										
MPKK5	**0.77**	**0.73**	**0.89**		**0.95**	**0.74**	**0.74**		**0.86**	**0.82**		**0.84**		**0.83**	**0.91**	**0.80**	**0.89**	**0.82**	**0.79**	**0.75**	**0.94**	**0.92**	**0.89**	**0.88**	**0.91**		**0.96**		**0.81**
MPKK6		**0.76**	**0.75**				**0.87**	**0.73**		**0.83**			**0.84**	**0.77**					**0.76**						**0.78**				**0.74**

[c]The threshold is 0.73 with a false discovery rate of 0.001 for indica rice lines.

Table S4. Polymerase chain reaction (PCR) primers used for quantitative reverse-transcription PCR assays

Gene name (GenBank accession number)[a]	RGAP code[b]	Forward primer 5'-3'	Reverse primer 5'-3'
MPK1	Os06g06090	TACATTCGCCAACTTCCTA	GCACCTTCAACTGTTATTCT
MPK2	Os08g06060	TGATCTTCACCACCTTCTT	ACATACTTCAATCCTCGTAGT
MPK3	Os02g05480	TGAAGGATATAATGATGCCAGTA	CTGAGGTGACTTGATTATCTGA
MPK4	Os06g48590	CATAGATGTCTGGTCTGTTG	GTTGTCAATGAACTCAATGTC
MPK5/MAPK5 (AF479883/AF479884)	Os03g17700	TCCGTGATGAACTTTGAG	GTCCATGTCGTTGTTGAA
MPK6 (EF174189)	Os10g38950	TTGCTACGAGGGCTAAAATATGTG	GGAACAAATTGCTTGGCTTCA
MPK7	Os05g49140	TAGGCAACATCAGGAAGAG	AGTAGTATTGTGAAGACCGTAT
MPK8	Os01g47530	CCACCAACCTATCAGCATA	CACCACCTTAGCATCCAT
MPK9	Os05g50560	GCAATGGAAGTGGCTCAG	GTAGAGTGAACAGTAGTAGACCTA
MPK10	Os01g43910	TTGAGCGTAGAAGAGTGA	CGGTGCCATTGATATAGTC
MPK11	Os06g26340	TTGCTGAGGTGTTGATTG	TTCTTGCCTTGTCATTCC
MPK12/BWMK1	Os06g49430	GCTTCCTCTATCCAAGTG	TTTCTCCTTTGCTGTAGTT
MPK13	Os02g04230	GCTAGTATCAGTGCTTCAAC	CTCATAGACAACCTCTTCCA
MPK14	Os05g05160	TTGGCGAATGCTGATTGT	AGATGGCGGTTGGAGTAT
MPK15	Os11g17080	TTGAAGAACATTATGCTAAGG	CATCCGAATATAACACAGAAG
MPK16	Os01g45620	GAGTTGTTAGGAATGGTGATG	AAGAGTGGCTTGTTCAGT
MPK17	Os05g50120	AACCTGACTTCTTCACTGA	GCCACAACACCATAACTC
MPKK1	Os06g05520	ATTCTGTTCATTCGTCTCTG	CATCATATCGGTTGCTTGT
MPKK3	Os06g27890	ATGCCTGATTCTGGACAA	GAACTGGTTCTGGTATTGATT
MPKK4	Os02g54600	GCTTCGGCCTGAGCATTCT	AGCAAATCGCGCACATGAG
MPKK5	Os06g09180	TCGGCCTCAGCATCCTAGAG	ATAGCAGATGGCGCACATGA

MPKK6	Os01g32660	CAAGCAACACAGAACGCACAT	GCAAGAGATCCACGGTCCAT
MPKK10-1	Os02g46760	TGGAGAAGGACTGGAGGAG	TAGATGCTCGTGTTCACTTGG
MPKK10-2	Os03g12390	GAACAGCTTTGTCCCGTGTGT	CCAAGAACGAACTTAAAAAACAAACA
MPKK10-3	Os03g50550	GGCTCTAATTAGGGAGAAGAGG	CGCATCTTGGAAGGGTACA
MPKKK1/OsEDR1 (KM103910)	Os03g06410	GAGTGAAGTGCGGATTATG	TTGGAGGACGAGTAACAG
MPKKK2	Os10g29540	GGTGTTGAAGATGATGCTATT	TCTGCTGGAATGAGTGTT
MPKKK3	Os02g32610	AGTGGTCAAGATGGCAAT	CAAGGAATAATCAAGTCTTCAATG
MPKKK4	Os02g12810	GCGCATTGGTCTAGGTTCATA	GATCTAGTGCAACACCCGATAA
MPKKK5	Os12g37570	CCTCAGTGGACATCACTTATCC	CTCGAAGTCTCTCCAGAAGTTG
MPKKK6	Os02g50970	CGTTGGTCCTTCTGATAGT	CCGAATGAATCTGATAGTGTATT
MPKKK7	Os06g12590	GCATCCTCAATGGTTGTC	CCAGGTTCCAAGTATTCGTA
MPKKK8	Os11g10100	CACCTTGCTCTAGCCCATTAC	GTTAGCTGCTCCAGCTGAATA
MPKKK9	Os02g44642	CTCTGACTGGTGGTAATG	GGCTCATCAAGACATCTC
MPKKK10	Os04g47240	CTTCAGGAGAGGGTGGTATCT	CGTTCAGATGGTGACAGCTTAT
MPKKK11	Os07g02780	AAGTTCCAAGATAAGAGTTCAA	TGTCGTATATTCGGCATTG
MPKKK12	Os09g39320	CCGAGATGAACCATCCAATGA	TGGGCAGGATTCAGATTACAC
MPKKK14	Os04g52140	GCAACAAGAACAGTATGG	TTATCGCCACTCTCAGAA
MPKKK16	Os04g35700	ATGATGAACGAGACAAGAC	GCTTTGGACCTTCAACTG
MPKKK17	Os09g37230	AATCACCTTCTCCACCATT	ATGCCAACCAACAACAAC
MPKKK18	Os03g55560	GCTAGATCTCCCGGTTCATTATC	GGACTGGACCTCAAACTCATC
MPKKK19	Os02g35010	TGGAGGTATTGTGAGGAG	ATGGTATCGCAGGACTAA
MPKKK20	Os07g38530	TGATTGATGGCGAGGTTA	GCAGTTATGGCAGTATGTAG
MPKKK21	Os07g25680	GAATTGTAAACAACACATTGAG	CCAGCACTGATCCATAAG
MPKKK22	Os03g49640	GAGTCTTGGCTGCACTGTATT	GGAATTTGAGGTCGTTCTCCTT
MPKKK23	Os12g40279	AGGTGGATGTGTTCTCCTTTG	TTGTTGACGATACCGCCTATG
MPKKK24	Os04g56530	CATCTGATACCGATTCTTGA	GCTTGTTCCTGTCTTCTT

MPKKK25	Os02g38080	CAATCTTGCCTCCTGAATCTCC	CCTTGGCTTGTCTTCCATCTT
MPKKK26	Os07g29330	TCTGGATGGAAGCACTGAA	AACGATGAGCACTGAGGAT
MPKKK27	Os03g43760	TCATCTGCTGTTGTCCAT	CACCACCTCATCCATATCA
MPKKK28	Os03g15570	GTGAGGAGGAGGATGATG	CATTCGGCGAGATGTAGA
MPKKK29	Os02g45130	TAGTGAGTATGGCGTTGA	GAGTGATGTGATGCTTGAT
MPKKK30	Os02g02780	TGAGGTCATTGAACATAAGC	GGTATCTTGCCAGTTAGC
MPKKK31	Os01g45380	AGGCACACTTGGTTACAT	GCATATCGCAGCAGTATATC
MPKKK32	Os08g12750	ATGCCTTGAGTGATGATG	TCGTAATCCTTGAGTGTCT
MPKKK33	Os02g07790	ATATGATAGGCGGTGGTG	TTATGGTAAGACGGCATGT
MPKKK34	Os05g50190	TTCTCAACGGCAGTCCATA	GGCATATCACAGCAGTATATCTC
MPKKK35	Os02g54510	GTGCCTTGACCCACCATATC	CCCTCCTTCATGCACTTTACTT
MPKKK36	Os05g01780	GAGACCGACAGTTCAGAA	CATTAGGCATATTACCACCAAT
MPKKK37	Os04g51950	GGACCTCAAGTCAGATAATC	CTTCAATCCTGGCAACTC
MPKKK38	Os06g45300	ATGCCGAGATAGAGGTTATG	TCAGTCTGTGGTCATTGG
MPKKK39	Os06g08280	CAATCTTCCACTCATCCA	CTTGTAACTGTCGCTTAATAG
MPKKK40	Os01g48330	CAGTCCTCTGTATGGATTATCA	TGACCTCAACATAACATTGC
MPKKK41	Os06g43840	AGGCACACTTGGTTACAT	GCATATCGCAGCAGTATATC
MPKKK42	Os03g60150	GGTTATTGTCAGCGAGTTAT	GGTCTCTGTGTATTATTCCATT
MPKKK43	Os06g50920	CCTTACTCAACTGTCTTCAC	CTTCTCCAACGCTTCTTC
MPKKK44	Os02g14530	CTGTTGGTATGGATTGTAAGTT	TAGCCTTGGATGAGAATGG
MPKKK45	Os06g43030	TGTCGGTGCTGGAGAAGTA	CTGAAGATCTTGGCGAAGGAG
MPKKK46	Os11g06140	ATGGTAAGTTCAAGAATGTTCA	AGTAAGAAGGCGTCTATCAA
MPKKK47	Os07g08750	GAAGAGGAGCAGCATTTG	CCTTGTACTGTGCCATCT
MPKKK48	Os01g01740	TCGCCGTGTATGATGTAT	CTTACTGCCGCATAATAGG
MPKKK49	Os05g44290	ACAGCGGCAAAGGAAATCTA	TTCAAGTTACCCGGTGTTCTC
MPKKK50	Os05g44290	CGTGCGGAACATCTACTT	AATACTCGTGCTGCTTGT
MPKKK51	Os01g54350	CACCAAGCAGGATGATAAG	TCAGGATGGAAGGAGAAC

MPKKK52	Os12g06490	GACACTCAAGCATAAGAACA	TTCATAGCCTTCATATTCACTT
MPKKK53	Os11g02305	CTCAGAACCACGACGATCAC	CTCTTGCTCCTCACTGGAATTA
MPKKK54	Os03g28300	GCCATATCCTTCACTCAGT	CTCTCACCTCCGACATAC
MPKKK55	Os01g50400	GCTGGATCCAAGTGCTCAA	CCACCGGAAGATTCGTTGAT
MPKKK59	Os12g41260	ATGCTGTGTTGTGGTAGA	GCTTCTTGTTCTTCCTGTAAT
MPKKK61	Os01g10450	CTGATGATGACGACAATGG	TGTGATAGAGGTGAGATAGAG
MPKKK62	Os01g50420	CGGAGGCGAAGAATTTCCTAT	GAGAAATGGGTGCTCCAGTAG
MPKKK63	Os01g50370	GGACGCTTTCGATCAGTCAA	CTGAACCAGCGCAGACTTTA
MPKKK64	Os07g39520	CTCCGTGAAGAAGGTGTA	CTTGTGGAAGCCGATGAT
MPKKK65	Os07g43900	GCGACCTTCATCAATACCT	AGTGATTGGCAGCAGAAT
MPKKK70	Os01g50410	AAGCGATCGAATCCTCCATTAG	GTCAGTATCGAACGGGTGAAA
MPKKK72	Os01g54480	TGACACTGGACATCTGAAG	ATTGCCTGGAGAAGTTACA
MPKKK74	Os01g66860	CGCCTTCTCATTTGGTTT	AGATTCCTCTGATGACTTCC
MPKKK75	Os02g39560	GAAGAGGAGAGGAAGTTACA	TATGCTATTGGCGTCTGA
Actin (X15865)		TGTATGCCAGTGGTCGTACCA	CCAGCAAGGTCGAGACGAA

[a]The names of MPKKKs, MPKKs, and MPKs are used according to Rao et al. (2010), Hamel et al. (2006), and Reyna and Yang (2006), respectively.

[b]RGAP (Rice Genome Annotation Project, http://rice.plantbiology.msu.edu/) locus identifier.
